# Estimating the government public economic benefits attributed to investing in assisted reproductive technology: a South African case study

**DOI:** 10.1016/j.rbms.2020.08.001

**Published:** 2020-09-04

**Authors:** Mark P. Connolly, Saswat Panda, Gitau Mburu, Thabo Matsaseng, James Kiarie

**Affiliations:** a University of Groningen, Department of Pharmacy, Unit of Pharmacoeconomics, Groningen, The Netherlands; b Global Market Access Solutions Sarl, St-Prex, Switzerland; c Department of Sexual and Reproductive Health and Research, World Health Organization, Geneva, Switzerland; d Reproductive Medicine Unit, Faculty of Medicine and Health Sciences, Stellenbosch University and Tygerberg Academic Hospital, Cape Town, South Africa

**Keywords:** infertility, public economics, in-vitro fertilization, live birth, resource allocation, fiscal analysis

## Abstract

Limited resources and high treatment costs are arguments often used in many public health systems in low- and middle-income countries to justify providing limited treatments for people with infertility. In this analysis, we apply a government public economic perspective to evaluate public subsidy for in-vitro fertilization (IVF) in South Africa. A fiscal model was developed that considered lifetime direct and indirect taxes paid and government transfers received by a child conceived by IVF. The model was constructed from public data sources and was adjusted for mortality, age-specific educational costs, participation in the informal economy, proportions of persons receiving social grants, and health costs. Based on current proportions of individuals receiving social grants and average payments, including education and health costs, we estimate each citizen will receive ZAR513,165 (USD35,587) in transfers over their lifetime. Based on inflated age-specific earnings, we estimate lifetime direct and indirect taxes paid per citizen of ZAR452,869 (USD31,405) and ZAR494,521 (USD34,294), respectively, which also includes adjustments for the proportions of persons participating in the informal economy. The lifetime net tax after deducting transfers was estimated to be ZAR434,225 (USD31,112) per person. Based on the average IVF investment cost needed to achieve one live birth, the fiscal return on investment (ROI) for the South African Government is 5.64. Varying the discount rate from 4% to 7%, the ROI ranged from 9.54 to 1.53, respectively. Positive economic benefits can emanate from public financing of IVF. The fiscal analytic framework described here can be a useful approach for health services to evaluate future public economic benefits.

## Background

Infertility is a disease that causes considerable suffering and hardship for those affected, and represents a significant public health burden (Dyer et al., 2016). Several studies have demonstrated that infertility can reduce quality of life in men and women, which can lead to profound emotional distress (Greil, 1997). For many individuals, infertility increases the risk of depression and anxiety compared with fertile persons (Greil et al., 2010), and can lead to prolonged lifetime conditions that include low self-esteem, emotional distress, isolation, social estrangement, hopelessness, anger, sexual and emotional abuse, and marital problems (Agostini et al., 2011; Rouchou, 2013; Schmidt, 2006; Valoriani et al., 2016). The consequences of infertility and childlessness in low- and middle-income countries (LMICs) can be even more pronounced due to cultural norms around fertility and gender roles in which the burden is often placed disproportionately on women (Cui, 2010; Rouchou, 2013). Estimates also suggest that low-resource countries experience higher rates of infertility compared with developed economies – a situation which is exacerbated by limited availability of treatment options (Ombelet and Onofre, 2019).

According to the International Committee Monitoring Assisted Reproductive Technologies, infertility is defined as the inability to conceive after 1 year of regular intercourse without contraception (Zegers-Hochschild et al., 2009). Estimating the prevalence of infertility can be challenging due to limited reporting, different data collection methods and the application of varying definitions of infertility. A large systematic review of data from 190 countries that used live birth as the outcome of interest found that 1.9% of women aged 20–44 years were unable to have their first child, and 10.5% were unable to conceive an additional child (Mascarenhas et al., 2012). Infertility is fairly common, with reported prevalence of 8–12.5% worldwide and up to 23% in sub-Saharan Africa (Larsen, 2000; Wellings et al., 2016). The most recent estimate reports that approximately 9.65 million women have primary or secondary infertility in sub-Saharan Africa (Mascarenhas et al., 2012). In South Africa, the birth rate has been decreasing steadily since the late 1960s (Burger et al., 2012) with the total fertility rate currently at 2.67 (STATSSA, 2015). In South Africa, the estimated need for assisted reproduction is 76,500 cycles per year. However, only 6.4% (Dyer and Kruger, 2012; SARA, 2014) of this need is met due to low access to assisted reproductive technology (ART) services. A significant proportion of these services are provided in the private sector and at a few public university hospitals. Invariably, access to ART involves costs to patients (Huyser and Boyd, 2013).

Economists have long recognized that procreation and rearing of children creates externalities extending beyond the family unit (Lee and Miller, 1990; Wolf et al., 2011). In developing countries, the economics of childbearing can influence the household in terms of labour and financial security in old age (Dyer, 2007). From the perspective of government and public finance, the size of birth cohorts can influence government spending and the collection of future taxes that can be generated from individuals (Auerbach et al., 1994). Adopting this analytic approach provides us with an established framework to evaluate infertility and fertility treatments, and those policies that influence access to fertility services and resulting externalities of children conceived by in-vitro fertilization (IVF) (Connolly et al., 2008, Connolly et al., 2010a, Connolly et al., 2010b). To inform the formulation of policies concerned with access to assisted reproduction, we apply the above fiscal analytic approach in a middle-income country, South Africa, to understand the consequences of public investments in IVF.

## Materials and methods

A public economic modelling framework consistent with established modelling practices was developed to evaluate the projected lifetime taxes paid and government transfers received from the perspective of the South African Government following the birth of an average child conceived by IVF with a base year of 2018 (Mauskopf et al., 2018). The model design is based on the generational accounting framework used by national governments to project the fiscal consequences of future government policies and spending, and the impact on future generations (Auerbach et al., 1994; Figari et al., 2015; Percival et al., 2007; Schofield et al., 2011). The public economic framework has been used previously to evaluate IVF investment costs in Europe, and this represents the first application of the approach in a middle-income country (Connolly et al., 2008, Connolly et al., 2011). In the context of health care, the framework can be used to evaluate individual health programmes and the likely impact on government accounts now and into the future based on investing in health programmes. The fiscal modelling framework is based on human capital economics often applied in the evaluation of health programmes, the difference being that the broader government perspective is reflected and not only the health service (Schofield et al., 2011). This approach enables governments to understand the short- and long-term fiscal consequences of health programme investments that change morbidity and mortality and can be used for setting health priorities by governments. The model was developed using Excel (Microsoft Corp., Redmond, WA, USA).

### Government transfers

We derived the average weighted costs paid by the South African Government to citizens for different social programmes (i.e. transfers) over their projected lifetime. All transfer payments received were weighted in relation to the proportion of people at each age and duration likely to be receiving the benefit and applying per person per annum cost paid by the national government (SASSA, 2019). The social grants included in the framework consisted of child support grants, foster child grants, care dependency grants, disability grants and old age grants (SASSA, 2019). The per pupil government spending on education adjusted for proportions of participants in each educational level at each age were also included (UNESCO, 2019). We applied per student educational transfers from pre-primary school education to tertiary school weighted for the proportion of state subsidy at each education level.

### Macroeconomic input parameters

To estimate the gross and fiscal position of a child requires factoring in macroeconomic parameters that can influence the individual input parameters and forecasts described here. The timeframe of the model was set to age 70 years, aligned with current life expectancy for South Africa (approximately 63 years), and retirement was set at age 65 years (WHO, 2019). To project income growth over the working life, we estimated the average salary growth rate over the period 2007–2016 based on income tax data reported to the South African Revenue Service (SARS, 2019). The wage growth of 7.17% was applied over the lifetime of the working ages. The analysis was adjusted for the age-specific proportion of the population active in the labour force (STATSSA, 2017). Future tax revenue was derived by applying age-specific wages to tax rates to projected future wages of a child born in South Africa. The effective tax rate on earnings was determined from the proportion of income tax paid in relation to gross earnings reported for each age category in the annual statistics (SARS, 2019). Indirect taxes were calculated using the incidence indirect tax which includes value added tax (VAT), excise tax and fuel levy (Valodia, 2019).

Social grants were also inflated in line with government spending projections (Kahn, 2017). The lifetime public health spending of a newborn child was also projected based on current spending patterns and adjusted for health cost inflation (Insight Actuaries and Consultants, 2019; STATSSA, 2019). The per person transfer payments and taxes paid in each year of life were adjusted for survivorship using the South African life tables. Consistent with cost-effectiveness guidelines from South Africa, a discounted rate of 5% was applied over the course of the model to reflect the present value of future transfers and tax receipts (DPME, 2019). As long-term financial projections are most influenced by the discount rate applied, a sensitivity analysis was conducted by varying the discount rate from 4% to 7%.

### Informal economy

Several calculations were included in the model to account for the proportion of informally employed persons who do not report income taxes but pay indirect VAT through consumption (Petersen, 2011). In 2011, more than two million persons worked informally in South Africa; in some provinces, such as Gauteng and KwaZulu Natal, informal employment accounted for 24.6% and 21.3% of employment, respectively (Petersen, 2011). Wages were adjusted for the proportion of people within the informal economy, where wages are often lower. For those participating in the informal economy, we considered that consumption spending would be taxed at the normal VAT rate. We conservatively assumed no changes in the proportion of those working in the informal economy over the lifetime of the model. In our analysis, children born to parents participating in the informal economy may also access social grants as eligibility is based on income, and those working informally with no reported income are more likely to meet eligibility criteria.

### IVF costs and outcomes

The average costs per live birth within public clinics in South Africa were estimated using the IVF treatment success rate from 2016 of 14.7% (SARA, 2014). The average cost per cycle was estimated by considering likely resources used for an IVF cycle, and then multiplied by the resource item cost and weighted for the frequency of use by couples in each fresh cycle. Treatment costs are based on resource use, and costs from a single public clinic in South Africa where the average cost per fresh cycle was estimated to be ZAR11,321 (USD785). The estimated costs per cycle used in our analysis are comparable with previously reported costs from public clinics (Huyser and Boyd, 2012). The average cost per live birth was determined by dividing the average cost per IVF cycle by the average IVF success rate to derive the average cost per live birth of ZAR76,984 (USD5338) (cost per cycle ZAR11,321/14.7%). This cost was used as the public investment cost for government from which future transfer payments and tax revenue would originate based on the estimated return on investment (ROI).

### Calculating lifetime taxes and transfers

Treatment of infertility with IVF is viewed as a human capital investment that will give rise to future (R) and public (E) transfers from the initial investment over the lifetime of the child. Furthermore, at each year of life, we derived age-specific net tax revenues from the government perspective based on deducting government transfers from gross tax receipts, and discounted these costs to the base year using the following equation:

$$\mathrm{NPV}=\sum_{t=0}^{T}\left(\frac{R_{t}-E_{t}}{{\left(1+r\right)}^{t}}\right)-K_{o}$$

where NPV is net present value, R_t_ is the sum of gross tax revenues (direct and indirect) accruing from the individual at age t, E_t_ is the sum of government expenditures on the individual at age t, r is the discount rate, T is life expectancy, and K_o_ is direct IVF costs paid by the South African public health system

The ROI from IVF investment is derived from [R_t_ *–* E_t_]*/*average cost per live birth.

Evidence from studies shows that where ART outcomes in South Africa are documented, multiple pregnancies are frequent (Botha et al., 2018). However, multiple pregnancies tend to be costly to health systems. It is therefore pertinent that ART services are geared towards elective single embryo transfer (eSET) to optimize the safety and cost-effectiveness of publicly funded ART. For instance, reimbursement policies to encourage single embryo transfers could be considered as an integral part of efficient publicly funded ART. Considering the move towards eSET, our model estimates the lifetime fiscal outcomes and ROI of a singleton pregnancy.

### Patient and public involvement

The development of the research question and the outcome measures were informed indirectly by the increasing rates of infertility, particularly in LMICs, and the lack of government funding for assisted reproductive services for patients suffering from infertility. Patients were not involved in the design, recruitment and conduct of this study. The study results will be disseminated to the public through journal publication, meetings and presentations.

## Results

**Figure 1:**
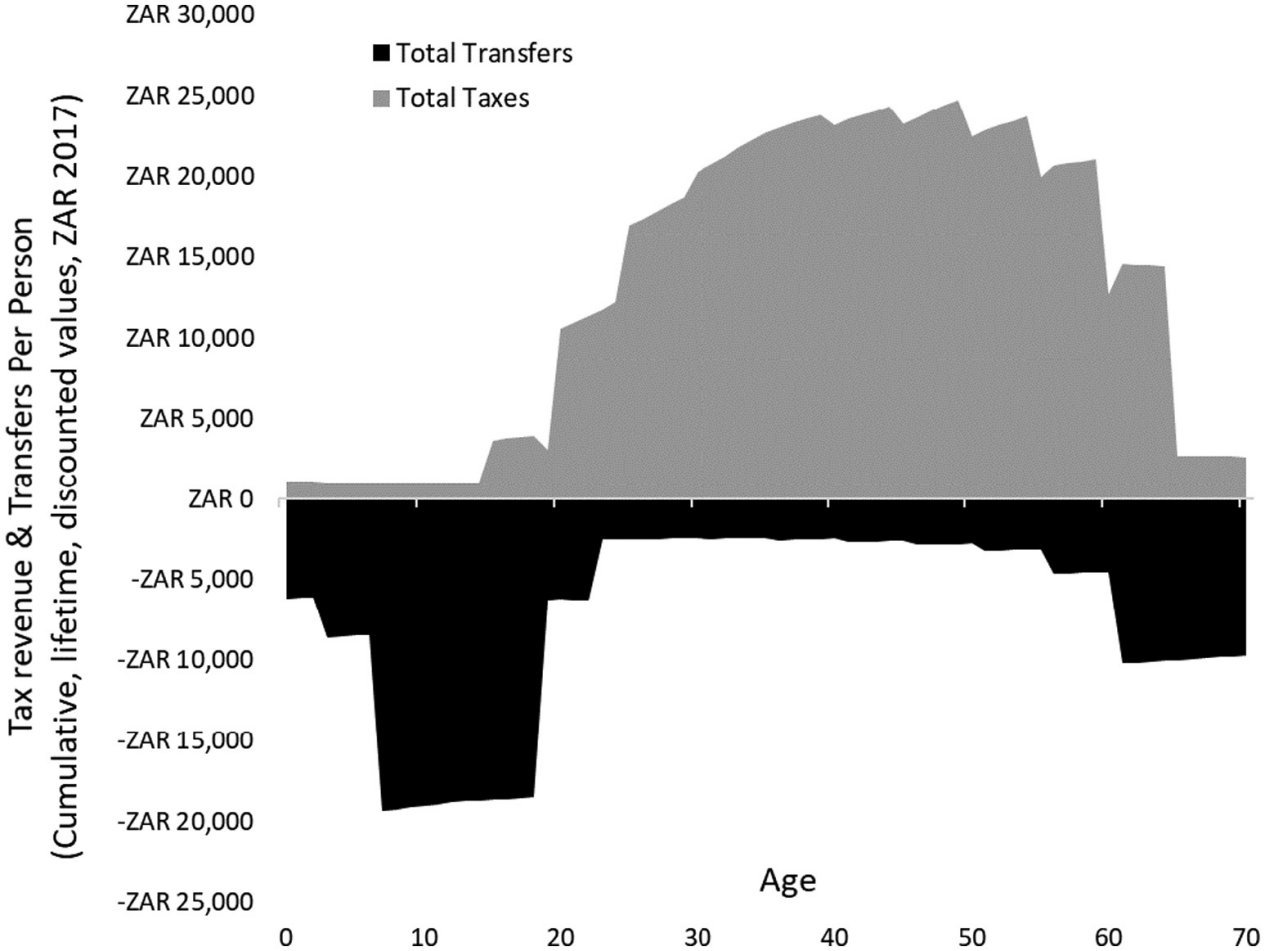
Average annual per capita transfers and taxes adjusted for mortality discounted at 5%.

The lifetime course of taxes and transfer payments is depicted in Figure 1. The weighted direct and indirect taxes derived from salaries are shown as positive transfers to government. The taxes transferred to government are adjusted for the proportion of individuals economically active at each age, and further adjusted for the informal economy and age-specific mortality. Once individuals enter the workforce, the age-specific annual taxes increase over the lifetime, and later decrease gradually as people transition into retirement. Transfer payments are depicted as costs to government and shown as negative. Peak transfers are received in early life years and during retirement. The weighted proportion of people that receive transfers over their working life years is also illustrated (Figure 1).

Central to the IVF fiscal model is understanding the lifetime discounted taxes and transfers of a child conceived by IVF. We estimate the weighted costs per social grant given per child, and the lifetime direct and indirect taxes paid (Table 1). Based on current proportions of individuals receiving social grants and average payments, including education costs, we estimate the lifetime average public benefits each citizen will receive to be ZAR513,165 (USD35,587) over different stages of their life based on current levels of need. From the age-specific earnings inflated for wage growth, we estimate direct and indirect taxes per citizen of ZAR452,869 (USD31,405) and ZAR 494,521 (USD34,294), respectively, which includes adjustments for the proportions of persons participating in the informal economy. The lifetime net tax after deducting lifetime transfers was estimated to be ZAR 434,225 (USD31,112) per person.

**Table 1:** Projected lifetime government transfers and tax payments discounted at 5%.

Social grants	Amount^b^
Education^a^ Old age grant Disability grant Care dependency grant Foster child grant Child support grant Health care^c^	ZAR 182,726 (USD12,671) ZAR 90,506 (USD6276) ZAR 30,293 (USD2100) ZAR 2691 (USD186) ZAR 4568 (USD316) ZAR 53,905 (USD3738) ZAR 148,475 (USD10,296)
Total transfers	ZAR 513,165 (USD35,587)
Direct tax Indirect tax	ZAR 452,869 (USD31,405) ZAR 494,521 (USD34,294)
Lifetime gross tax	ZAR 947,390 (USD65,699)
Lifetime net tax (gross tax – transfer payments)	ZAR 434,225 (USD30,112)

a Weighted for the proportions attaining each grade level applied to per pupil costs.

b Converted from XE.com (ZAR to USD) 1-year average.

c Excludes costs of in-vitro fertilization treatment.

The ROI attributed to IVF therapy in the base-case discount rate of 5% was 5.64. A sensitivity analysis around the discount rate at 4%, 6% and 7% for the ROI calculation, total transfers, gross taxes and net taxes was also conducted where the results were found to be sensitive to the discount rate applied (Table 2).

**Table 2:** Total transfers, gross tax and net tax at varying discount rates.^a^

Discount rate	4%	5%^c^	6%	7%
Total transfers^b^	ZAR693,633 (USD48,102)	ZAR513,165 (USD35,587)	ZAR399,300 (USD27,690)	ZAR323,989 (USD22,468)
Gross tax	ZAR1,427,789 (USD99,014)	ZAR947,390 (USD65,699)	ZAR641,098 (USD44,459)	ZAR442,404 (USD30,679)
Net tax	ZAR734,156 (USD50,912)	ZAR434,225 (USD30,112)	ZAR241,798 (USD16,768)	ZAR118,415 (USD8212)
Return on investment	9.54	5.64	3.14	1.53

a Converted from XE.com (ZAR to USD) on 28 February 2020.

b Includes lifetime education, old age grant, disability grant, care dependency grant, foster child grant, child support grant, and health care.

c 5% discount rate applied in base case.

## Discussion

Various economic frameworks can be used to evaluate health and investments in health care, representing a broad range of perspectives and value frameworks (Doshi and Willke, 2017; Mauskopf et al., 2018). Infertility and its treatment are unique amongst all health interventions because the successful implementation of treating infertile couples results in a live birth. In economic terms, the resulting child represents an externality attributed to treating infertility that is a consequence of successful implementation of treatment. Conventionally, economic assessments of health focus on the benefits to the individual, and there is no means by which to capture what the future child may represent – unless one focuses solely on the quality of life that the child may bring to the parents. Consequently, focusing on quality of life attributed to fertility treatment and future success or failure in terms of quality of life assigns no economic value to the resulting child and likely undervalues the intervention. In contrast, the field of public economics regularly applies frameworks by which the child can be valued as a future member of society (Connolly et al., 2008; Wolf et al., 2011). Fiscally, the child represents both costs and benefits for government that can be estimated based on established actuarial relationships and per person lifetime taxes and transfers. In our analysis, we reflect an IVF live birth from the government perspective to understand how governments can be economically impacted by children born from IVF. In this respect, we performed a cost–benefit analysis that focuses on the lifetime taxes paid by a future child and the access to public benefits over their lifetime. Within tax financed health systems, this approach seems particularly valid as public money is used to treat infertile couples; hence future taxes will arise from public investments.

Medical treatments for infertility often face funding challenges as health systems struggle to pay for more immediate health needs. While the goals of health systems are to improve health, increase life expectancy and provide equitable access to services, treatment of infertility is often seen as a luxury that few health systems are willing to fund. However, from the perspective of health system sustainability, treatment of infertility and the addition of future citizens that contribute to tax financed pay-as-you-go health systems will help to ensure that there are people in the future available to pay taxes to fund the system. This might suggest that funding for IVF should be considered in the context of longer-term financial stability, and contemplate the fiscal relationships and demographics that underlie financing of health systems and the consequences of a changing tax base. The analysis described here illustrates the benefits of investing in IVF and the lifetime fiscal consequences for government. While individual returns are positive, when this approach is applied to a cohort of children conceived by IVF, the results illustrate the broader macro level impact for government of policy changes that influence access to care (Connolly et al., 2011).

All health conditions can have fiscal consequences for government that vary depending on the severity of the condition and the age of onset when the health event occurs. Health events that prevent people from working, dying prematurely or working below their optimal output translate into lower lifetime earnings, which translates into less tax revenue for government (Schofield et al., 2011). Furthermore, people with disabilities will require income support enabling them to maintain a standard of living (Schofield et al., 2011). Understanding this relationship enables us to think about health conditions and their impact on government in a different environment, especially within tax financed health systems that rely on a younger and healthier population. The approach applied here has been used to evaluate a range of different health conditions including paediatric and adult vaccination, mental health and smoking cessation policies, suggesting that the framework outlined here could be applied to a range of healthcare systems (Connolly et al., 2017). Furthermore, as this approach applies a lifetime framework and illustrates fiscal flows between citizens and state, it can be used for priority setting within health systems to establish how illness and health investments will influence government transfers.

The World Health Organization (WHO) promotes the use of research evidence in developing health policy, clinical guidelines and health technology assessments (WHO, 2014). However, policy formulation and decision-making processes by ministries of health regarding access to technologies are routinely challenged by the lack of adequate evidence on public health interventions, including those related to infertility (Rutstein and Shah, 2004; Tso et al., 2011). Having adequate evidence and data on the burden and cost-effectiveness of interventions for infertility can inform policy makers in thinking through different investments for infertility services, including ART. This is particularly relevant because WHO recognizes infertility care as an integral component of sexual and reproductive health and rights.

The results described here highlight the public economic consequences of children conceived by IVF using the human capital approach in a middle-income country. However, it is important to contextualize these findings in relation to broader policies on family planning and prevailing fertility rates within a country. In recent years, South Africa has experienced a significant demographic transition, largely due to changes in fertility (Geyer and Mosidi, 2019). A range of biomedical, social and structural factors, such as child support grants, education, government population policies, employment-related migration and changing social norms related to marriage, have played a role in changing fertility desires in South Africa where couples are having fewer children and often later in life; however, the current total fertility rate is still greater than replacement level (Burger et al., 2012; Geyer and Mosidi, 2019; Moultrie and Timæus, 2003). Conversely, in countries with birth rates below replacement level (i.e. < 2.1 births per woman), with real concerns over age-dependency ratios and sustainability of public finances, there can be an economic imperative for each additional child, and IVF can be seen as an extension of pronatalist family policies. Moreover, how can these results be interpreted in the broader context of family planning and available contraception within a country which may lead to fewer children being born, and should this represent a future fiscal loss or gain? To some extent, this analysis may overinterpret the value of each additional child because factors such as future job opportunities and access to resources could diminish in the presence of too many children, with consequent diminishing of societal and fiscal return. The fiscal reality is likely somewhere between these two extremes. Certainly, each life has some measurable economic value, regardless of whether a child is conceived by IVF or naturally. However, the underlying economics will be influenced by the social and familial environment to which the child will be born. Furthermore, it is important to recognize the underlying dynamics of choice and the ability of individuals to control the timing and desired family size, especially in countries that fund both family planning and infertility treatments. In this regard, fulfilling unmet needs for fertility treatments, IVF may be seen as an important intervention that facilitates the right of every woman/couple to bear children or achieve reproductive desires, especially in countries with high documented unmet need of assisted reproduction. By doing so, IVF will help countries to achieve a demographic dividend that can help stimulate economic development (Bloom et al., 2009).

Although previous studies have explored the public economic consequences of IVF funding to inform public funding of assisted reproduction (Connolly et al., 2010a, Connolly et al., 2010b), this study is the first to explore the application of a fiscal framework in an middle-income country undergoing social and economic transformation. Central to economic development in LMICs is the role of taxation and the ability of countries to fund their own public services (Besley and Persson, 2013). Recognizing that international agencies and grants can only do so much for economic development, eventually nations need to generate domestic revenue through taxation to fund public programmes. Compared with other LMICs, South Africa has a fairly established taxation system that has been working diligently to reduce tax evasion, limit the informal economy, raise taxes to redistribute wealth and reduce economic inequality, and achieve steadily increasing annual tax revenues (SARS, 2019). Although not all LMICs have such a well-structured tax system as found in South Africa, this work can instill a sense of urgency and importance to be gained through taxes, which is the cornerstone of taxation and development literature (Besley and Persson, 2013).

There are several weaknesses to the fiscal model framework described here. Firstly, the long-term fiscal projections applied here are based on a set of present micro- and macroeconomic conditions that will certainly change over time. As is standard practice in modelling, these factors have been held constant. The model is also limited by the availability of quality data. Some variables, such as the distribution of social grants, rely on data from 2014, the most recent published report for this type of data. Other elements of the model, such as the adjustment for informally employed workers, were subject to assumptions because this area of the South African economy is not well documented. While it is understood that a proportion of people who are not recorded as being formally employed are informally employed, it is difficult to know the specifics of this population regarding annual income, income growth, consumption and exact proportions of people employed informally by age strata. Additionally, South Africa is a diverse country with major wealth disparity and inequality which can distort input parameters and, consequently, fiscal projections described here. Therefore, results will vary within the country. As the framework here depicts an average life course for a child born today, it is difficult to obtain data which reflect the diverse economic conditions which can influence model conclusions. Furthermore, the focus of this analysis has been on the positive fiscal consequences of those achieving a successful live birth. However, many more people will not succeed in achieving a live birth, resulting in additional co-morbidities and costs such as depression which are not accounted for here. Nevertheless, our findings show that apart from potentially positive psychological benefits to infertile parents, IVF may deliver positive fiscal impact to the government under the set of parameters applied today.

An additional weakness exists regarding the socio-economic profiles of those attending public and private clinics in South Africa, and the likely fiscal trajectories of the children conceived in these clinics. Private sector and privately funded IVF predominate ART in South Africa. Current data on assisted reproduction are largely generated from middle-class households that seek ART from the private sector. The very poor, who form a significant majority in South Africa, are unable to obtain assisted reproductive services in the private sector, and therefore their data may not be captured in routine data and research. This is particularly relevant given that a significant majority of the middle-class citizens who seek ART often get into poverty due to catastrophic expenditure. Although they are willing to pay, they are not necessarily able to afford it. Evidence shows that many of these middle-class households struggle to recover economically following expenditure on ART in the private sector and end up in long-term financial hardship (Dyer et al., 2017). Even where patient co-payment for one cycle of ART was provided in a university hospital, catastrophic expenditure affected one in five households, mainly the poorest (Dyer et al., 2013).

The health sector in South Africa is largely bimodal, with a significant role of both the public and private sectors. Publicly funded universities also accept single out-of-pocket payment for one cycle of ART. As South Africa moves ahead to implement a national health insurance scheme, we suggest that IVF could better fit into this model through integrated reproductive health services similar to the Scandinavian model, where eligible candidates qualify for three cycles of IVF.

Considerable attention is often focused on the costs of treatment and those factors that influence access to treatment (Ombelet and Onofre, 2019). Our analysis applies the costs per cycle adjusted for treatment success, and this is used as an investment for government that has long-term fiscal consequences. Whilst the costs of IVF used in our model are based on a single clinic, what is clear is that the cost per cycle and cost per live birth are minimal compared with all other costs considered over the lifetime of a child conceived by IVF. Whilst costs paid by patients are an important consideration, we prefer to focus on the future public economic value, where we demonstrate that ZAR947,390 in lifetime discounted future gross taxes can emanate from a single child conceived by IVF.

## Conclusion

The results described here illustrate the potential future public economic value attributed to children born through ART in South Africa. These results are sensitive to prevailing economic conditions, particularly the likely discount rate applied in the analysis. We believe the framework described here can help inform resource allocation decisions in LMICs; however, the results should be interpreted in the context of other health priorities and disease burden.
